# Proof of concept of a static approach to determine mechanical tissue properties during tumor surgery

**DOI:** 10.1186/s42490-025-00100-9

**Published:** 2025-11-03

**Authors:** Max Jäger, Katja Uhrhan, Christine Mucha, María Alejandra Guzmán Alfaro, Hartmut Witte

**Affiliations:** Biomechatronics Group, Technische Universität Ilmenau, Max-Planck Ring 12, 98693 Ilmenau, Thuringia, Germany

**Keywords:** Tumor surgery, Palpation, Young's modulus, Force measurement, Indentation

## Abstract

The mechanical properties of tumor tissue differ from those of healthy tissue. Therefore, surgeons palpate accessible surgical sites to determine tumor boundaries prior to resection. However, palpation is not possible during minimally invasive surgery, so instrumented palpation is required instead. This study investigates the suitability of an engineering method that combines mechanical object scanning and indentation to determine Young’s modulus of soft, tissue-like materials. To establish a defined reference, we tested our concept on silicone phantoms containing stiff tumor-like inclusions. We used a sensor consisting of a load cell connected to a rigid probe with a spherical indenter tip. Young’s modulus was calculated by measured force, indentation depth, and indenter geometry. These results were compared with those of a palpation experiment on the same specimens, conducted with surgeons. Validation results reflect the accuracy of the method. Error in estimation of Young’s modulus is: soft material 6.7%, stiff material 44.9%. Repeatability is high, with a standard deviation < 7%. By scanning a phantom and creating a stiffness image, we were able to identify the location and shape of the inclusion more clearly than experienced surgeons could using manual palpation. Looking ahead, the prospect of miniaturizing the presented technique for localizing tumor boundaries during surgery seems promising.

**Supplementary Information:**

The online version contains supplementary material available at 10.1186/s42490-025-00100-9.

## Introduction

A key surgical principle in tumor resection is “as much as necessary, as little as possible”. This is especially critical in head and neck (ENT) surgery and neurosurgery, where even minimal removal of healthy tissue can result in significant functional impairment or reduced quality of life for the patient. Therefore, accurate intraoperative identification of tumor boundaries is essential to optimize surgical outcomes.

A promising approach to delineate tumor margins involves the assessment of mechanical tissue properties, such as stiffness and damping, which are in combination expressed as mechanical impedance. While tactile feedback of a material’s impedance through manual palpation can be useful when the tumor is directly accessible, this method becomes infeasible in neurosurgery and increasingly limited in ENT surgery as access depth increases. In these scenarios, instrumented (e.g., robotic) palpation using sensor technology offers a potential solution and a means to obtain quantitative data from an otherwise subjective task. A wide range of measurement principles and sensor concepts for the determination of mechanical tissue properties have been discussed in literature, e.g. ultrasound-based [1, 2], optical [3, 4] and force sensing, the latter integrated into the grippers of surgical tools [5]. While these approaches provide useful results, they require expensive equipment and extensive data processing, which is time consuming. Our goal is to measure mechanical tissue properties using indentation and comparatively simple calculations to provide results intraoperatively within short time. Several approaches based on direct contact, using measurements or estimates of force and tissue deformation, have already been reported in the literature.

Recognizing the need for objective measurements and smaller probes that allow in-vivo measurements of mechanical tissue properties, Francis et al. [6] developed a sensing system with two degrees of freedom (DoF) for prostate palpation. While the sensing tip itself is small, the entire system is still comparatively large. Smaller complete measurement systems can be found in [7–11]. Choi et al. [11] present a handheld foot ulcer detection probe that uses a spherical tip to indent the tissue and measure the resulting force. Because the probe is handheld, there is still room for error due to inconsistent pressure applied to the foot by the operator. A similar handheld probe is described in Beccani et al. [10]. They propose a wireless palpation probe that uses a barometric pressure sensor to determine the elastic modulus of tissue by measuring the depth of indentation using an external static magnetic field that also facilitates localization of the probe. Shaikh et al. [9] present another small, pen-like probe to obtain objective and quantitative information about tissue stiffness. They aim to improve oral cancer diagnosis by determining the modulus of elasticity from the ratio of two parallel forces, resulting from different materials interfacing with the tissue. McKinley et al. [8] propose a sensor that can be attached to a DaVinci surgical robot’s needle driver tool to provide palpation as the probe slides across the tissue surface. The sensor consists of a linear compression spring and a magnetic encoder that allows force measurements from deflection data. With a similar goal, Scimeca et al. [7] use a capacitive sensor array [12] to investigate palpation strategies. They show that with the right strategies, information can be gained from less data or from data of lower quality. Konstantinova et al. [13] investigate force modulation during manual palpation. They use their findings to implement autonomous robotic palpation and show an improvement over static, point-by-point measurements. Although the sensors developed by Beccani et al., Shaikh et al. and McKinley et al. are comparatively small, they all require perpendicular contact with the tissue under investigation. The sensor system described by Kalwa [14] avoids this by sucking the sensor onto the surface of the tissue. Mechanical properties are measured by displacing the tissue with saline solution at a known pressure and measuring the water volume through its electrical properties. They implement both Young’s modulus and viscosity measurements with that system.

Previous studies have primarily concentrated on the technical realization of various sensing approaches, often without quantitatively evaluating their performance in clinically relevant tasks such as tumor margin detection. In contrast, this work presents a proof-of-concept for a palpation tool specifically designed for the estimation of tumor boundaries in tissue-like materials, including variations in depth. The developed laboratory setup enables precise force measurements and systematic evaluation of the method’s accuracy and provides reproducibility in identifying differences in material stiffness associated with tumor margins. Comparison with manual palpation data collected in an exploratory study highlights the enhanced performance of our approach. The primary contribution of this work lies in the technical validation of a sensing method capable of delivering quantifiable and reproducible results aimed at a challenging surgical context.

## Materials and methods

### Young’s modulus derived from measurements of contact mechanics

The basis of our measurement approach is the calculation of Young’s modulus from contact mechanics [15–17]. For the indentation of an elastic half-space with a much stiffer sphere, the following equation can be used [15].

1 $$\:{E}={F}\left(1-{\nu\:}^{2}\right)\frac{3}{4{R}^{\frac{1}{2}}{\delta\:}^{\frac{3}{2}}}$$

where *E* - Young’s modulus (elastic modulus), *F* - force normal to the contact surface, *ν* - Poisson’s ratio, *R* - radius of the indenter tip, *δ* - indentation depth. Using Eq. (1), *E* can be calculated from measurements of *F* and *δ*. Both *R* and *ν* are assumed to be constant. We chose *ν* = 0.5 because we only consider static indentation in a steady state and our samples are silicone phantoms.

### Tissue phantoms

Silicone tissue phantoms were manufactured according to the process described by Garg et al. [18]. For the measurements described in the following sections, four phantoms were used: one stiff, one soft, and two with a stiff inclusion in a soft surrounding material. The inclusions were cylindrical and star shaped, respectively. The materials used were silicone rubber: Soft: Ecoflex 00–20 (Smooth-On Inc., USA); stiff: PREMIUM 230 Silicone Transparent (S u. K Hock GmbH, Germany). The phantoms were cast in plastic Petri dishes. For the inclusions, the stiff material was cast in smaller Petri dishes (diameter 30 mm, height 5 mm) or cut to the desired shape from larger casts. The phantoms with inclusions were produced by first casting a homogeneous, but flatter soft phantom. After curing, the inclusion material was laid on top of the soft material and more soft, uncured material was added until the inclusion was covered completely. The phantoms have a diameter of 85 mm and a thickness of roughly 15 mm.

For the stiff phantoms, we calculated Young’s modulus from the shore hardness (A-30) using the equations by Lutz [19] (Eq. (2)) and Gent et al. [20] (Eq. (3)).

2 $$\:{E}=2\left(1+\nu\:\right)\cdot\:\frac{0.07515{H}_{A}+0.549}{\left(4.1+3.9\cdot\:{e}^{-1.397\cdot\:h}\right)-\left(0.395h+0.315{h}^{2}\right)}$$

3 $$\:{E}=\frac{{g}}{100}\cdot\:\frac{56+7.66{H}_{A}}{2.67r\cdot\:\left(254-2.54{H}_{A}\right)}$$

The Eqs. (2) and (3) deliver 1.14 MPa and 1.15 MPa, respectively, using *H*_*A*_ = 30 for shore hardness A-30, *ν* = 0.5, *h* = 0.025 ∙ (100 - H_A_) (from Stommel et al. [21]), *r* = 0.515 mm (from Gent et al.). Since these equations only apply for shore A, Young’s modulus of the shore 00 phantoms was estimated from experimental data in the literature. Young’s modulus of the soft phantoms is assumed to be about 51 kPa, based on four studies [22–25] (standard deviation of the mean values from this literature: 9.54 kPa).

Both materials are assumed to be linear-elastic, which is reasonable for deformations below 10% for biological tissue [26]. For the silicone rubber, we chose the limit of 10% deformation to reduce potential influences of strain softening or hysteresis [27].

### Measurement setup

**Fig. 1:**
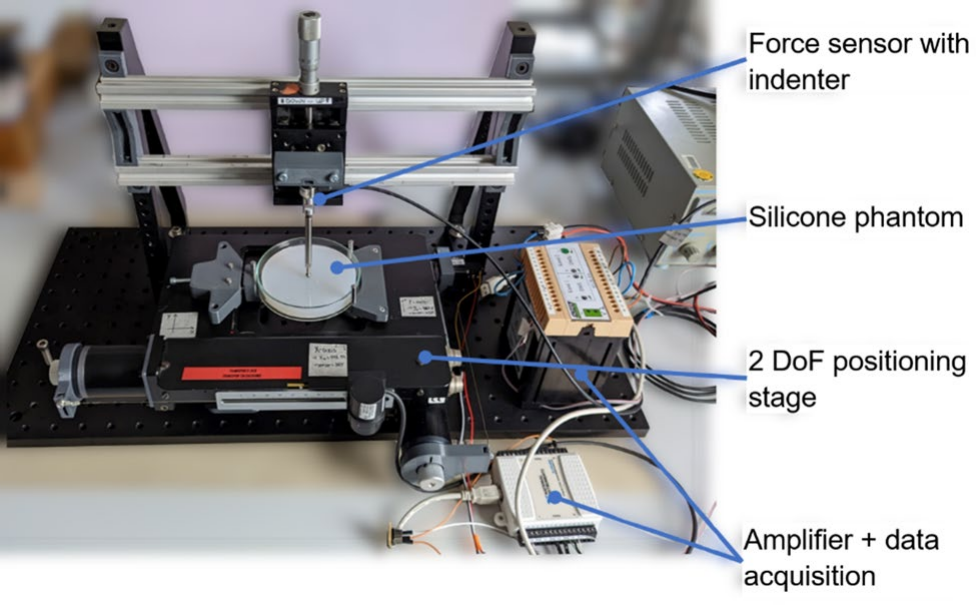
Measurement setup for force and indentation depth acquisition

We used the following measurement setup to acquire force and indentation depth data (cf. Figure 1).

**Fig. 2:**
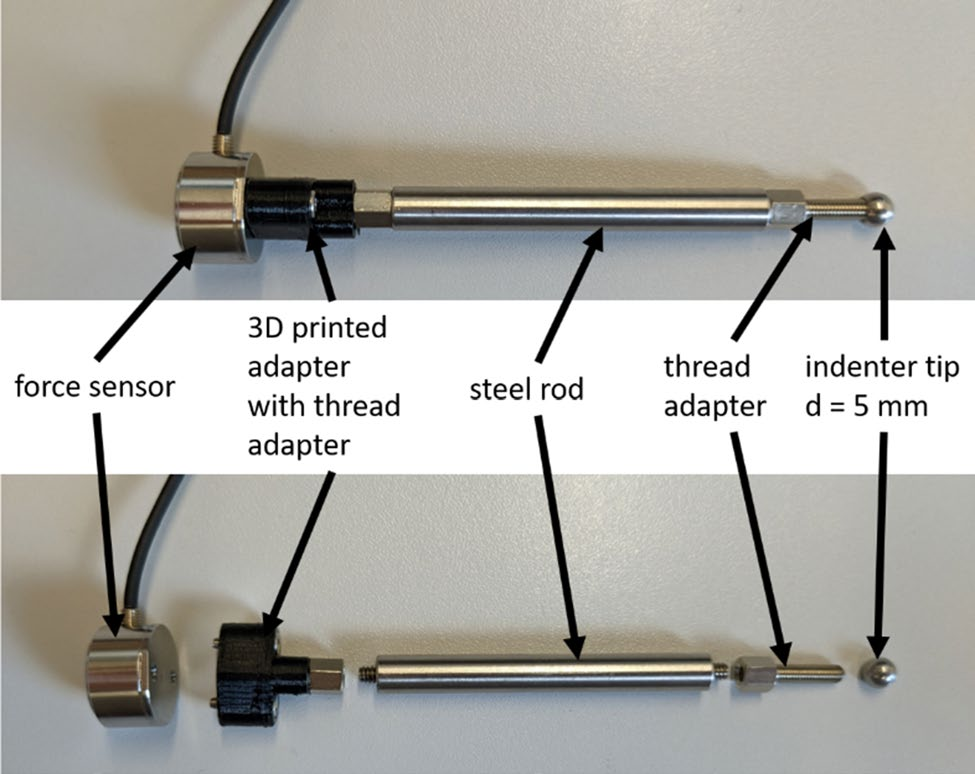
Force sensor and intenter tip assembly. The thread adapters have UNC #4–40 threaded holes and M3 threaded bolts

Force measurements were acquired using a one-axis strain gauge sensor (Sauter CO 1-Y5, KERN & SOHN GmbH, Germany) and an amplifier (SG-2K-KS, Althen GmbH Mess- und Sensortechnik, Germany). The sensor was referenced using known weights. With a linear fit of the reference measurements (*R*^*2*^ > 0.999), a slope of 0.7658 N/V was determined. The sensor offset voltage is measured at the beginning of each measurement session and subtracted from the sensor’s signal before conversion to force. A spherical indenter tip (diameter 5 mm) is attached to the sensor using a steel rod (4” = 101.6 mm, ER4 - Cage Assembly Rod, Thorlabs Inc., USA). Adapters with UNC #4–40 threaded holes and M3 threaded bolts were used to attach the indenter tip to the rod and the rod to the sensor (cf. Figure 2).

To position the sensor relative to the tissue phantom, we use three perpendicular linear stages. The phantom was placed in a 3D printed, spring-loaded clamp attached to a table that can be moved in the x and y axes. The x and y axes are driven by stepper motors using ball screws with 4 mm pitch. The stepper motors are controlled using a microcontroller (Arduino Uno, Arduino S.r.l., Italy) and two stepper motor drivers (TMC2209, BIGTREETECH, PR China).

The force sensor is attached to a linear stage (OWIS GmbH, Germany) with a fine-thread screw mounted on a gantry. During the measurements, the indentation depth is manually read from the scale on the screw. The position of contact between the indenter tip and the phantom surface is defined as the starting point for the indentation (*δ* = 0 mm) and is visually determined by the investigator for each measurement point.

The amplified force sensor signal is read using a USB data acquisition box (NI USB 6008, National Instruments, USA) and a LabVIEW (National Instruments, USA) measurement program. In the user interface of the LabVIEW program, the measurement parameters can be specified: The diameter of the indenter tip, the slope to calculate force from the measured voltage, and the duration of the measurement for each measurement position. After measuring the force sensor offset, the indenter tip can be moved to the desired x-y position by controlling the stepper motors. The force signal is sampled at 1 kHz for the set measurement duration and stored in a text file.

The acquired data are processed offline in a Python (Python 3.11.0, Python Software Foundation, USA) script. Data from each measurement point are averaged over the measurement duration. Young’s modulus for each point is calculated using Eq. (1) and the averaged data.

### Validation measurements

#### Precision and accuracy

To validate our approach, we measured the same point on two homogeneous tissue phantoms (one stiff: Shore A-30, one soft: Shore 00–20) multiple times (*N* = 10). For all measurements, an indentation depth of 0.5 mm was used. As reference moduli, the mean values of the respective ranges described above for stiff and soft silicone material were used to calculate the relative error of the measured values to estimate the accuracy of our setup. Using the repeated measurements, we calculated the standard deviation of the estimated Young’s modulus values for each phantom as a measure of the method’s precision.

#### Tissue discrimination

**Fig. 3:**
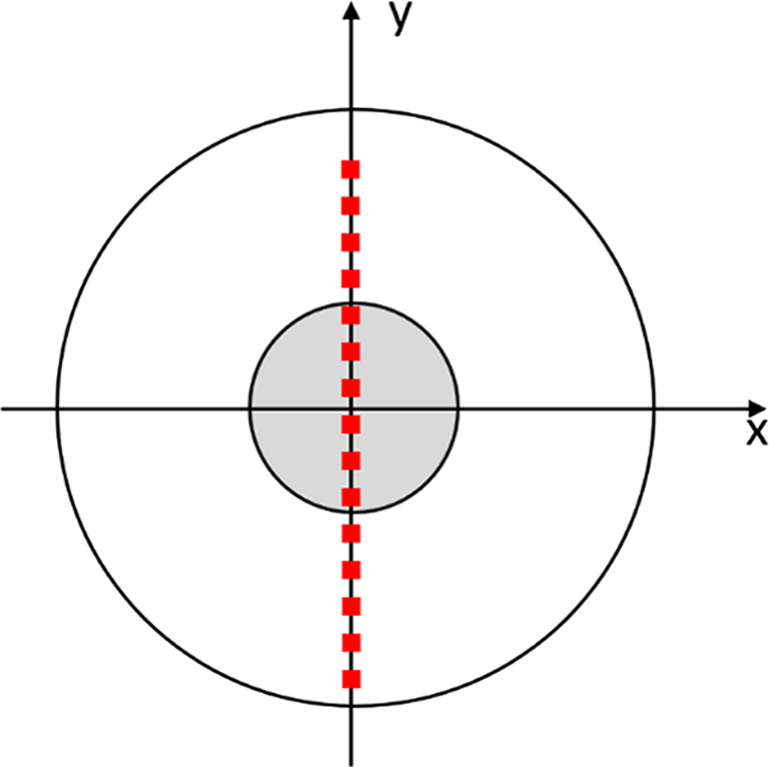
Schematic of the line scan measurement points on a silicone phantom with a circular inclusion

To investigate the performance of our approach, a line scan was performed on a soft tissue phantom with a stiff inclusion in the center (cf. Figure 3). At 15 points along the diameter of the phantom (in the y direction), we measured Young’s modulus with an indentation depth of 0.5 mm. The measurement was performed twice on the same phantom with a cylindrical stiff inclusion, once with the inclusion close to the surface at around 1 mm depth and once with about 7 mm of soft material above it. This was achieved by simply flipping the phantom upside down.

We used the Mann-Whitney-Wilcoxon test [28, 29] (implemented in R version 4.3.1 [30]) to check for a significant difference between the calculated Young’s modulus values inside and outside the inclusion region.

### Palpation experiment

We conducted a small experiment with experienced surgeons from ENT and neurosurgery departments (*N* = 6, 1 female) to investigate the typical palpation behavior of surgeons and their ability to discriminate tissue based on stiffness alone. The study was conducted in accordance with the Declaration of Helsinki and approved by the Ethics Committee of Technische Universität Ilmenau (Reference No. 2025-06-209_3.10_FoA_Uhrhan). Written informed consent was obtained from the subjects.

**Fig. 4:**
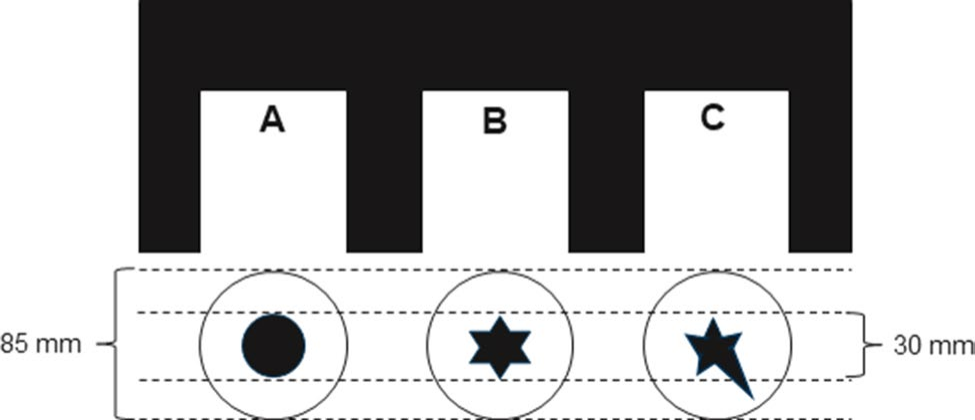
Schematic of the palpation experiment. Surgeons were asked to palpate three different silicone phantoms covered with a cardboard box (top). Each phantom had a differently shaped stiff inclusion in the center that the surgeons had to draw (bottom)

The surgeons were presented with three tissue phantoms with a stiffer inclusion (A-30) in soft silicone (00–20) and asked to examine them by palpation. Each phantom had a differently shaped inclusion in the center (see Fig. 4). During the palpation test, the phantoms were covered by a cardboard box with holes for the hands. The surgeons were then asked to draw the shape of the inclusion after blindly palpating the phantoms.

To compare our method’s performance in detecting an inclusion’s shape with results from manual palpation, we conducted surface scans on a tissue phantom with a star-shaped inclusion. Two surface scans were performed with different spacing of the measurement points and different areas of the phantom. For the first scan, roughly half of the phantom’s surface was scanned using a 2 mm grid. For the second scan, one of the star’s tips was scanned in a 1 mm grid. These scans are compared to the surgeons’ drawings from the experiment described above to get a first impression of performance of our method compared to the surgeons’ capabilities to estimate inclusion shape from stiffness as determined by manual palpation.

## Results

### Validation measurements

#### Precision and accuracy

Repeated measurements on the same tissue phantom show overall good precision and accuracy (see Table 1). For both materials, the standard deviation of our measurements is below 7% of the mean (soft: 3.4%; stiff: 6.7%). Taking into account the range of Young’s modulus values for the soft material found in literature (see section “Tissue Phantoms“ above), our indentation tests slightly overshoot the maximum value found (60 kPa vs. our 64 kPa, relative error 6.7%). For the stiff material, the mean estimated Young’s modulus is far below the reference value (relative error 44.9%).

**Table 1:** Validation measurement results for homogeneous “healthy” and “tumor” silicone phantoms. Mean reference modulus values are calculated from literature values for soft [22–25] and stiff [19, 20] materials

	Mean measured modulus / kPa	Mean reference modulus / kPa
Soft (shore 00–20)	64 ± 2	51
Stiff (shore A-30)	631 ± 4	1145

#### Tissue discrimination

**Fig. 5:**
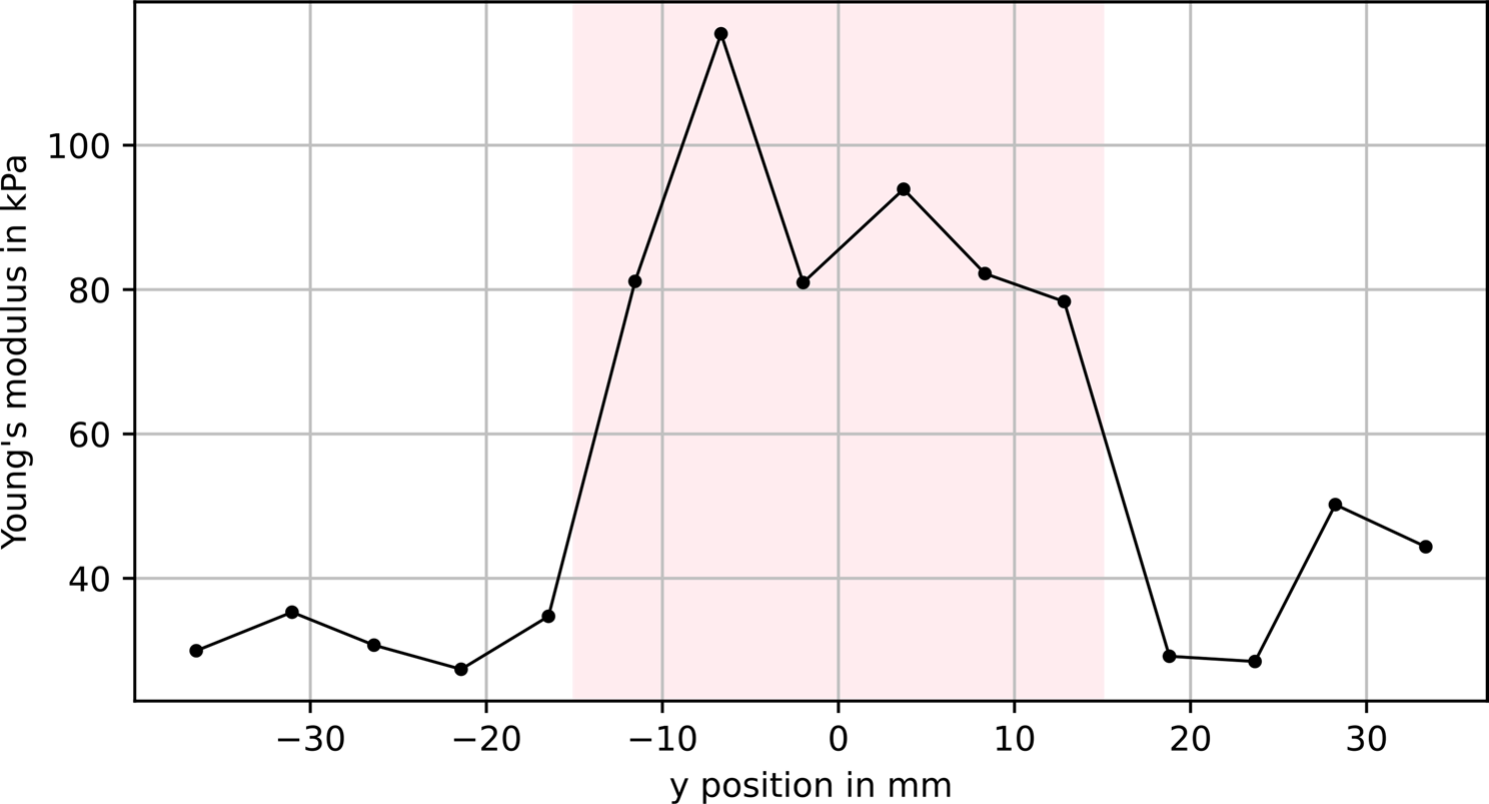
Young’s modulus calculated from measuring at 15 points along the y direction. Indentation depth: 0.5 mm. The inclusion was close to the surface (1 mm depth). The pink area marks the actual position of the inclusion

**Fig. 6:**
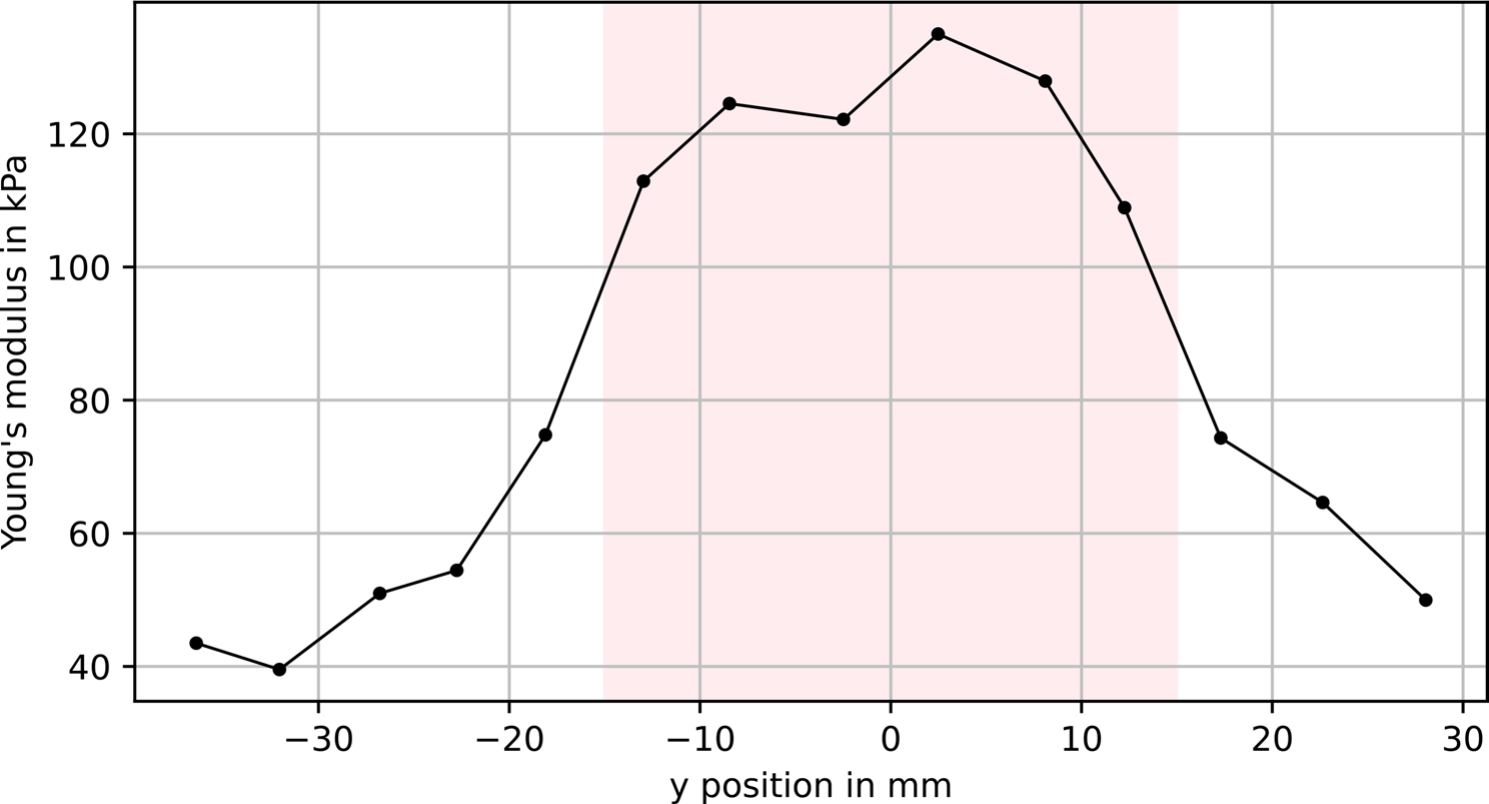
Young’s modulus calculated from measuring at 14 points along the y direction. Indentation depth: 0.5 mm. The inclusion was 7 mm below the surface. The pink area marks the actual position of the inclusion

The line scan as described above was performed twice on the same phantom with a cylindrical stiff inclusion, once with the inclusion close to the surface and once with about 7 mm of soft material above it. Figures 5 and 6 show the resulting elastic modulus profile for the inclusion at the surface and below 7 mm of soft material, respectively.

For both measurements, Mann-Whitney-Wilcoxon tests were performed. In both cases, the difference in Young’s modulus between the points above the stiff inclusion and the points above the soft material is significant, with a significance level *α* of 5% (see Table 2).

**Table 2:** Statistical evaluation (Mann-Whitney-Wilcoxon test) of the line scan data for measurements with the inclusion close to the surface and below 7 mm of soft material

	*p*	W	Cohen’s d	*N*_soft_	Mean_soft_ / kPa	*N*_inclusion_	Mean_inclusion_
Close to surface	0.002	0.0	-4.978	9	34 ± 7	6	88 ± 13
Below soft material	0.004	1.5	-2.523	8	56 ± 12	6	121 ± 9

### Performance assessment

**Fig. 7:**
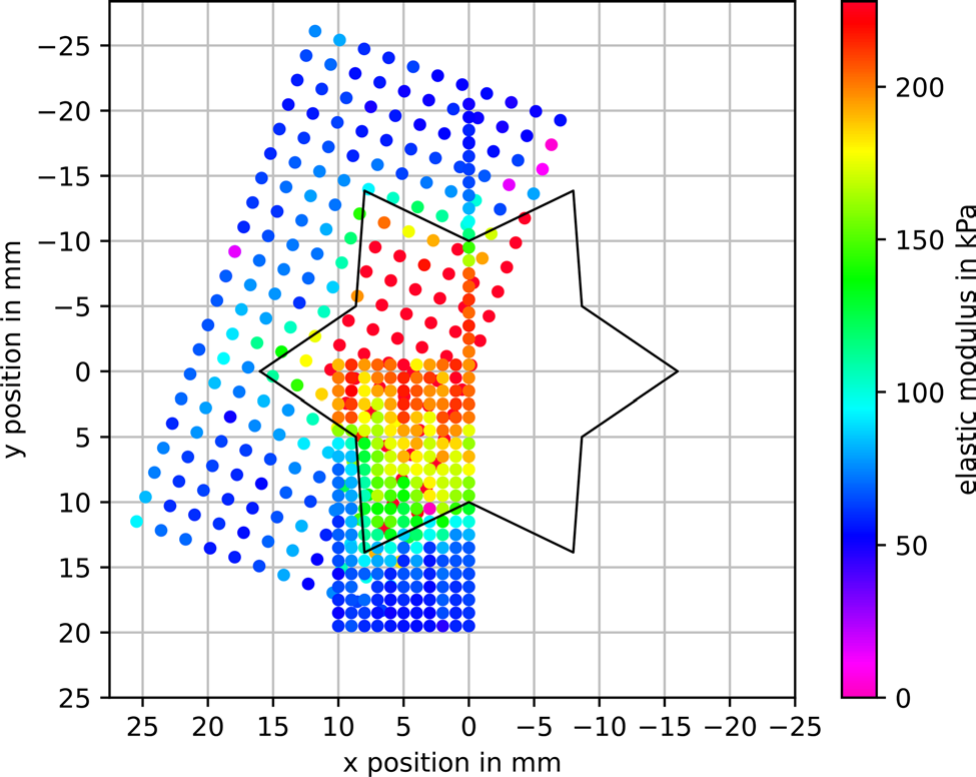
Overlaid results from two surface scans of a tissue phantom with a stiffer, star-shaped inclusion. The black outline marks the position and shape of the inclusion, based on measurements on the phantom

Figure 7 shows the overlaid measurements of the tissue surface scans. Note that the coarser measurement’s data was rotated to compensate for an offset during data acquisition. It is also important to note that the first two columns of the coarser measurement points (counted from the bottom right corner of the rectangular grid) contain erroneously high modulus values due to the inaccurate tissue contact determination (see discussion below). The first column of fine measurement points shows good agreement between the two measurements. Overall, the shape of the inclusion can be estimated from both measurements. The contour can be discerned much more clearly in the higher-resolution measurement. Using the Mann-Whitney-Wilcoxon test (omitting the erroneous measurement points), a significant difference can be found between measurement points within the inclusion outline (*n*_*in*_ = 180, cf. Figure 7) and outside (*n*_*out*_ = 270) (*p* < 10^− 6^, *W* = 584, Cohen’s *d* = 3.311).

**Fig. 8:**
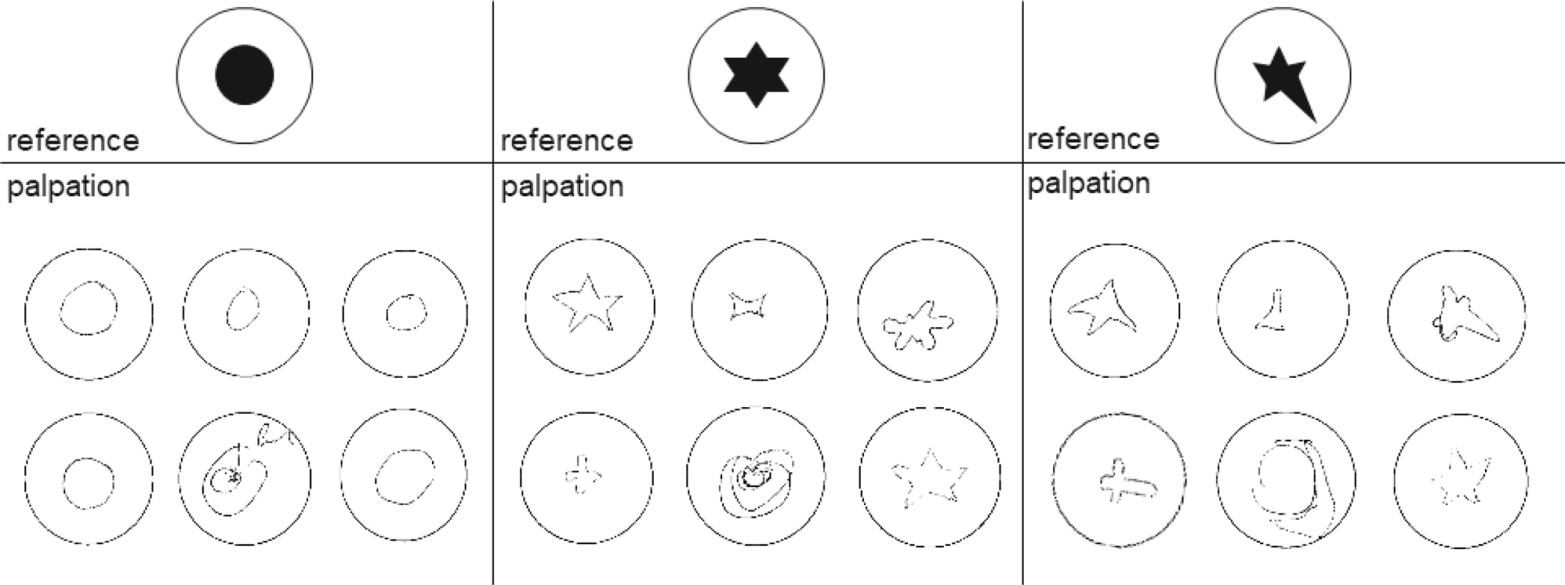
Results of the palpation experiment. Surgeons were asked to blindly palpate silicone phantoms with stiffer inclusions and draw the respective shape of the inclusions

Figure 8 shows the drawings made by the surgeons during the palpation experiment. While the circular inclusion can be considered correctly detected, both star-shaped inclusions proved to be more difficult. When comparing the robotic palpation results with the surgeon’s drawings, it seems safe to assume that the proposed method provides better capabilities for inclusion shape detection than manual palpation.

## Discussion

### Validation of the measurement setup

**Fig. 9:**
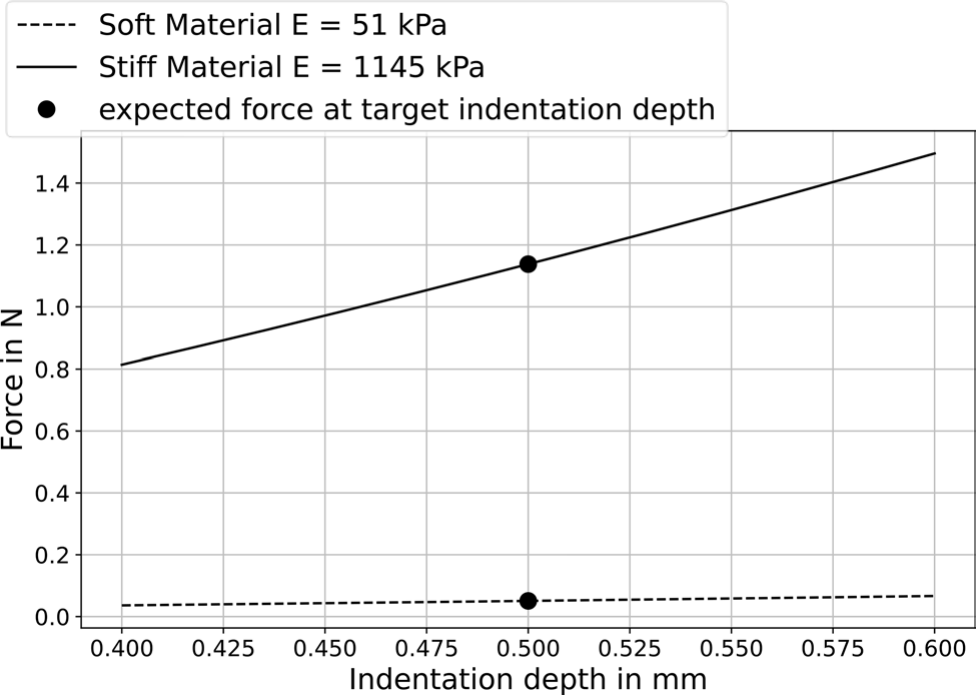
Expected force vs. indentation depth for soft and stiff material. The dot marks the target indentation depth used in our investigations and corresponding expected forces according to contact mechanics calculations (Eq. (1)). Errors in actual indentation depth lead to deviations in the measured forces from the expected values. As Young’s modulus is higher in stiffer material, incorrect indentation depth results in a larger error in estimated elastic modulus for stiffer material than for softer material

For both soft and stiff homogeneous tissue phantoms, the proposed setup shows good reproducibility. The calculated Young’s modulus was more accurate for the soft material than for the stiff material (relative error 6.7% for soft material, 44.9% for stiff material). This is mainly due to indentation depth errors. According to Eq. (1), the stiffer the material being tested, the greater the effect of an incorrect indentation depth on the calculated Young’s modulus. As can be seen in Fig. 9, small deviations in indentation depth have little effect on the measured force in soft materials, whereas in stiffer materials the measured force depends more strongly on the indentation depth. The combination of the target depth value and measured force from an inaccurate indentation using Eq. (1) leads to degraded Young’s modulus accuracy.

The actual indentation depth is highly dependent on the detection of contact between the sample and the indenter tip. Our approach to visually identify this contact is most likely the reason for the deviations observed in our measurements. While the indentation error impacts the accuracy of the estimated Young’s modulus, the reproducibility is still good (standard deviations below 7% of the respective mean: soft: 2 kPa; stiff: 4 kPa). Consequently, visual contact identification seems to be inaccurate, but reproducible.

### Limitations

Regarding speed and reproducibility, automation of the tip movement in and around all three spatial axes would be beneficial. Especially the indentation axis is prone to error. Limitations of the method of visual contact detection are discussed below. A more human factor is the tendency to select whole scale divisions as the point of tissue contact. This is facilitated by the lack of binary information regarding “contact” or “no contact”.

For minimally invasive surgery in ENT and neurosurgery applications, the indenter cannot be positioned perpendicularly to the tissue surface in most cases. Thus, measurements of torque are more useful, which could be achieved using a combined force and torque sensor. Detection of tissue contact and determination of indentation depth face a similar problem: In a laboratory setup, visual contact confirmation is a valid, albeit slow, approach. Error estimation and the maximum allowable uncertainty of the indentation depth are discussed in Appendix A. During surgery, higher speeds and automated detection of indentation depth, also during dynamic (sliding) tissue contact, would be beneficial. Possible solutions from the literature are discussed in the following section.

Another limitation of the measurement principle is the requirement for linear-elastic sample behavior to correctly measure Young’s modulus. This requirement is particularly relevant for future measurements on biological tissue, as excessive penetration depth could lead to tissue damage, which would distort the material behavior and thus lead to incorrect measurements. In order to assume linear-elastic behavior in biological tissue for a correct calculation of Young’s modulus, the indentation depth must not exceed 10% of the thickness of the tissue sample [26]. For future applications on biological tissue, the indentation depth must therefore be feedback-controlled so that the 10% limit is not exceeded and no tissue damage occurs.

## Discussion of the proof of concept

Despite the limited accuracy described above, the proposed system is able to detect a stiff inclusion in soft material and produce statistically significant different modulus estimates. This is true for both a circular inclusion close to the surface and 7 mm below the surface, and for more complex shapes, i.e., a star-shaped inclusion. Under idealized conditions, the measured stiffness distribution should exhibit sharp edges at the transition from soft to hard material. As one could expect, the additional soft material above the inclusion leads to a softening of the transition, similar to the blurring of an image through frosted glass. Nevertheless, the method is able to provide a 2D stiffness image that gives a clearer assessment of the inclusion’s shape than manual palpation was able to achieve.

For the intended application of tumor detection in ENT and neurosurgery, the proposed method provides a means to collect tissue stiffness information in a sterilizable way: Since only the indenter tip itself needs to be in contact with the tissue, the force sensor can be covered during surgery. This way, only the removable indenter (cf. Figure 2) needs to be sterilized. For a long indenter, where the sensor is placed outside of the patient’s body, applications in ENT and neurosurgery rarely permit vertical tissue contact. Therefore, a sensor that can detect torques and lateral forces is required. Possible solutions are 6-axis force/torque sensors, as used in Konstantinova et al. [13]. Investigations regarding continuous measurements are necessary. Especially, determining the indentation depth during e.g. sliding palpation as described in McKinley et al. [8] on surfaces that are not completely flat is a remaining challenge. Options to address this problem include, but are not limited to, capturing the surface before automated indentation is performed [31], using a transparent indenter tip to infer indentation depth from contact area [32] and measuring tool deflection while controlling indentation force [33].

The presented measurement setup can be used for laboratory experimentation or ex-vivo tissue analysis. However, parallel application with resection to preserve healthy tissue requires a more miniaturized, easier-to-handle probe. This is true for both handheld application and if the probe should be attached to a surgical robot. For handheld use of such a probe, the tracking unit should not hinder the surgeon’s movements. For indenter tip pose measurement during surgery, fiber-based tracking of position and orientation could provide a solution [34]. If the probe is attached to a surgical robot, the robot’s pose information can be used in conjunction with binary tissue contact status.

Using more elaborate palpation strategies as described in Konstantinova et al. [13] or Scimeca et al. [7] promises to improve inclusion detection performance. Gwilliam et al. [35] also compare the human performance in detecting stiffer inclusions in softer surrounding material. Their system provides comparable detection capabilities to the human subjects, however, requiring less indentation depth and pressure. Their investigations compare the sensor and fingertip in the same test setup: The sensor resp. fingertip remain static, and the indentation is imposed by a positioning system. This approach prohibits the additional information gain from the enteroception of the finger movements in manual palpation. The inclusions under investigation are smaller than the fingertip and manufactured from polyoxymethylene. In contrast, we compare more natural manual palpation to a sensor on a positioning system, using comparatively soft inclusions much larger than the fingertip. The results however similarly show improved stiffness distribution capturing capabilities when using the sensor.

## Conclusion

In this paper, we investigate a method to determine the stiffness of tissue from force and indentation depth measurements for use in tumor surgery. The approach was validated using silicone tissue phantoms, demonstrating both feasibility and reproducibility. The method yielded significantly different Young’s modulus estimates between stiff inclusions and surrounding soft material (*p* < 0.004 across all measurements). To evaluate the system’s clinical relevance, we compared its ability to delineate tumor phantom boundaries with manual assessments performed by surgeons. The results indicate improved accuracy in identifying inclusion shapes using the proposed method. This highlights the potential of our system to support intraoperative decision-making. We also discussed current limitations and outlined necessary improvements for integration into clinical practice, particularly in ENT and neurosurgery. Measurements on ex-vivo biological tissue samples are planned to assess whether the performance on silicone tissue phantoms is transferable to the clinical application. To maximize the significance of these measurements, samples including tumor tissue are needed. These efforts will form the basis for adapting the system to future in-vivo applications.

## Appendix A: Error estimation and allowable indentation depth uncertainty

Main contributors to estimation errors for Young’s modulus can be derived from Eq. (1). Errors from radius *R* and Poisson ratio *ν* can be considered negligible: *R* is constant throughout all measurements, and given the static nature of our measurements with relatively short measurement times (0.5 s) and the materials used for the phantoms (silicone rubber), the assumption is that the material behaves incompressible and thus ν = 0.5 is reasonable. Thus remain force *F* and indentation depth *δ*. Inspecting all force data used for this paper, the averaged standard deviation of all measurements is below 0.48 mN. Using this information, the maximum uncertainty of indentation depth *δ* can be calculated using error propagation. The limit is determined by the stiffness difference between materials that should be distinguishable by the system. In our case, based on our measurements on the phantom with a stiff inclusion below 7 mm of soft material, we assume the moduli to be *E*_*soft*_ = 50 kPa and *E*_*stiff*_ = 100 kPa.

A1 $$\:\frac{\partial\:{E}}{\partial\:{F}}=\left(1-{\nu\:}^{2}\right)\frac{3}{4{R}^{\frac{1}{2}}{\delta\:}^{\frac{3}{2}}}$$

A2 $$\:\frac{\partial\:{E}}{\partial\:\delta\:}=-\frac{9{F}\left(1-{\nu\:}^{2}\right)}{8{\delta\:}^{\frac{5\:}{2}}{R}^{\frac{1}{2}}}$$

A3 $$\:{u}_{E}=\sqrt{{\left(\frac{\partial\:E}{\partial\:F}\cdot\:{u}_{F}\right)}^{2}+{\left(\frac{\partial\:E}{\partial\:\delta\:}\cdot\:{u}_{\delta\:}\right)}^{2}}$$

Equations (A1) and (A2) are partial derivatives of Eq. (1) to *F* resp. *δ* and Eq. (A3) is the resulting combined uncertainty of *E* as described in [36]. We select a range of three uncertainties (so that roughly 99.7% of the values are included) to ensure a clear distinction between the materials. Using Eqs. (A1) - (A3), the maximum allowed uncertainty of *δ* can be determined as the intersection point of *E*_*soft*_ + 3*u*_*E*_ and *E*_*stiff*_ – 3*u*_*E*_. This yields *u*_*δ*_ = 0.037 mm. This is dependent on the Young’s modulus values that should be discriminated and has to be recalculated for other use cases.

## Supplementary Information

Below is the link to the electronic supplementary material.

Supplementary Material 1

## Data Availability

The measurement data can be found in the electronic supplementary material.

## Code availability

The code can be found in the electronic supplementary material.

## Abbreviations

ENT: Ear Nose and Throat
DoF: Degree of Freedom
USB: Universal Serial Bus
